# Bacteria-Based Microdevices for the Oral Delivery of Macromolecules

**DOI:** 10.3390/pharmaceutics13101610

**Published:** 2021-10-03

**Authors:** Zhenping Cao, Sisi Lin, Jinyao Liu

**Affiliations:** Shanghai Key Laboratory for Nucleic Acid Chemistry and Nanomedicine, Institute of Molecular Medicine, State Key Laboratory of Oncogenes and Related Genes, Shanghai Cancer Institute, Renji Hospital, School of Medicine, Shanghai Jiao Tong University, Shanghai 200127, China; caozhenping@renji.com

**Keywords:** oral delivery, microdevices, biologicals, bacteria, biomedicine

## Abstract

The oral delivery of macromolecules is quite challenging due to environmental insults and biological barriers encountered along the gastrointestinal (GI) tract. Benefiting from their living characteristics, diverse bacterial species have been engineered as intelligent platforms to deliver various therapeutics. To tackle difficulties in oral delivery, innovative bacteria-based microdevices have been developed by virtue of advancements in synthetic biology and nanotechnology, with aims to overcome the instability and short half-life of macromolecules in the GI tract. In this review, we summarize the main classes of macromolecules that are produced and delivered through the oral ingestion of bacteria and bacterial derivatives. Furtherly, we discuss the engineering strategies and biomedical applications of these living microdevices in disease diagnosis, bioimaging, and treatment. Finally, we highlight the advantages as well as the limitations of these engineered bacteria used as platforms for the oral delivery of macromolecules and also propose their potential for clinical translation. The results summarized in this review article would contribute to the invention of next-generation bacteria-based systems for the oral delivery of macromolecules.

## 1. Introduction

Biologics, also known as biomacromolecular pharmaceuticals, including peptides, proteins, antibodies, polysaccharides, and nucleic acids, produced from various biological systems, such as microorganisms, mammalian cells, and animal- or human-derived tissues via biotechnological means [1]. These biomacromolecules have been widely used for the prevention, diagnosis, and treatment of human diseases, such as tumors, AIDS, cardiovascular and cerebrovascular diseases, and hepatitis [1,2,3,4,5,6,7]. Currently, approximately 30 percent of all the U.S. Food and Drug Administration (FDA) approved drugs are biomacromolecules [8]. Among them, proteins and peptides take up the majority of these therapeutic biomacromolecules [8,9,10,11]. Owing to their clinical success and rapid improvements in commercial values and market shares, biologicals have been recognized as one of the most promising areas in drug research and development in the 21st century.

Although biologicals provide high specificity and activity thanks to their large and complex structures, there are problems and obstacles that need to be solved for the use of these drugs as most of them suffer from low stability, short half-life, and limited penetration across biological barriers [2,8]. In order to improve their treatment efficacy, the preferential method for the use of biologicals is injection, which is able to promote drug absorption, rapidly increase the blood drug concentration and enable accurate dosing [8,12]. However, conventional parenteral administration inevitably has problems with pain, patient incompliance, needle sickness, needle-stick injuries, and risks of systemic infections [13]. In contrast, the oral delivery of macromolecules drugs is a more convenient and non-invasive approach, with significant ameliorations in patient compliance and reduction of economic cost [1,8]. Unfortunately, the oral delivery of biomacromolecular pharmaceuticals faces biological barriers and microenvironmental insults encountered along the gastrointestinal (GI) tract [1,10]. For instance, the GI tract presents harsh environments with low pH in the stomach as well as high levels of bile salts and enzymes in the intestine for digestion and processing, which can severely reduce the efficacy of oral biomacromolecular drugs [1,14]. Therefore, innovative delivery strategies and systems of oral therapeutic macromolecules are highly desirable to overcome these challenges, with an overarching goal to increase bioavailability.

**Figure 1:**
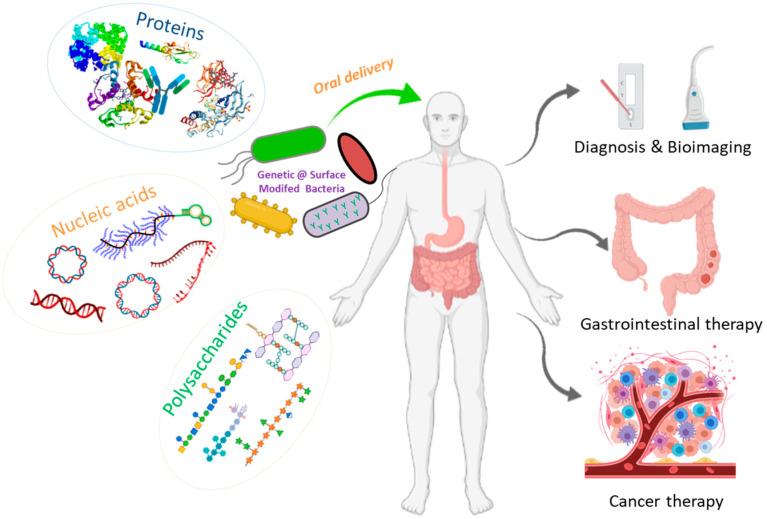
Schematic illustration of the oral delivery of therapeutic biomacromolecules by bacteria-based microdevices for disease diagnosis and treatment.

In recent years, many researches have been focusing on the development of medical devices including microneedle-based pills, nanostraws, microjets, hydrogels, intestinal patches, and bacterial therapeutics, which are capable of overcoming these biological barriers and orally delivering a wide range of biologics [15,16,17,18]. These new delivery devices are designed to enable the oral dosing of biomacromolecules and improve their bioavailability, with increased patient adherence and reduced pain and other side effects [1,10,15,19,20]. Bacteria have been investigated as drug delivery microdevices due to their living characteristics that are able to carry macromolecular drugs via genetic engineering [21]. Moreover, various therapeutics can be attached to bacteria through physicochemical modifications [22,23]. Given the ability to colonize specific positions, for example disease sites, bacteria-based systems have been designed to release drugs preciously and continuously in the targets of interest. Compared to conventional drug delivery systems for the oral delivery of macromolecules, bacterial microdevices exhibit the advantages of the in situ production of biologicals, long-term colonization in the intestine, targeting ability, and versatility to load diverse drugs [24,25]. In this review, we mainly focus on bacteria-based microdevices and summarize their current progress and future prospects for the oral delivery of biomacromolecules (Figure 1). We firstly introduce the different kinds of biomacromolecular drugs prepared and delivered by bacteria and bacterial derivatives. Then, current technologies for the modification of bacteria-based therapeutic macromolecules are described, with an emphasis on the strategy of genetic engineering. Lastly, biomedical applications of these bacteria-based microdevices as well as their future prospects in this field will be discussed.

## 2. Biomacromolecules-Loaded Oral Bacterial Microdevices 

Bacteria play extensively, yet important, roles in our lives both directly and indirectly [26]. With the development of interdisciplinary research on bacteria and their applications, numerous bacteria have been widely administrated as oral probiotics or bacterial therapeutics for treating various diseases including cancers, diabetes, inflammatory bowel diseases (IBDs), and pathogenic infections [27,28,29,30,31,32,33]. Due to their unique characteristics, such as genetic manipulation, rapid proliferation, and targeting specificity to disease sites, bacteria have successfully expressed different kinds of biologics through biological technologies and exhibited extremely promising potential to be utilized in bioimaging, diagnosis, and therapy [34,35,36]. Currently, the reported therapeutic biomacromolecules that are delivered by bacteria mainly include proteins, glycans, and nucleic acids. In this section, we focus on the introduction of the types and biomedical functions of these biomacromolecular drugs.

### 2.1. Therapeutic Proteins/Peptides

Proteins have specific and dynamic functions including forming receptors and channels, transporting molecules, and catalyzing chemical reactions [37,38,39]. Diseases may be caused, when proteins in the host body present mutations or abnormal concentrations [1,11]. Since their ability to serve a set of functions that are more specific and tolerable than small molecules, proteins are attractive to be used as therapeutics [11]. For several decades, therapeutic proteins have been considered as an important class of pharmaceutical biomacromolecular drugs [11], which encompass natural or engineered versions of large proteins, peptides, and antibodies [40,41]. With the rapid development of molecular biology and biotechnology, increasing numbers of recombinant proteins have been emerged and employed as pharmaceuticals, playing different clinical roles in healthcare (Table 1) [2].

**Figure 2:**
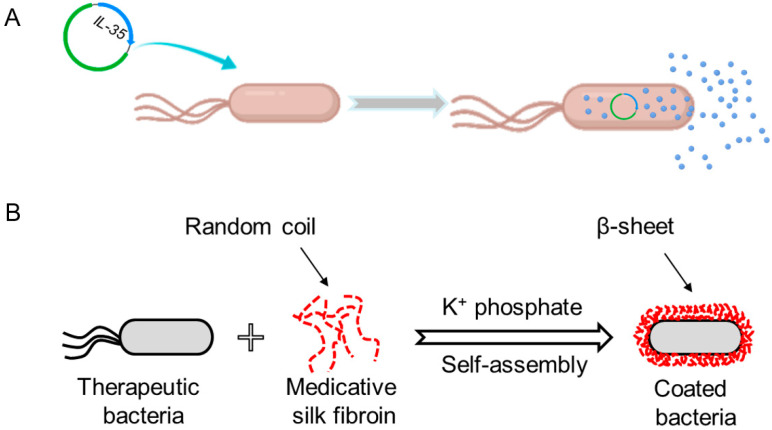
Engineered bacteria for the oral delivery of therapeutic proteins. (**A**) Oral delivery of heterogeneous interleukin (IL)-35 expressed by genetically modified bacteria. Adapted from [47], published by Springer, 2019. (**B**) Oral co-delivery of therapeutic silk fibroin and probiotics through self-assembly on the surface of bacteria for synergistically enhanced biotherapy. Adapted with permission from [50]; published by Wiley-VCH, 2021.

To date, hundreds of proteins have been approved by the FDA for clinical applications, and there are many more in the pipeline [10,11]. However, because of the limitations of recombinant bacterial systems, lots of proteins are expressed as inclusion bodies, which can be used functionally only after renaturation processes [42]. At the time of clinical trials, human insulin, as the first recombinant therapeutic protein, was generated by combining separately pre-produced chain A and chain B via chemical conjugation [43]. Subsequently, recombinant insulin is predominantly expressed in the large scale by *Escherichia coli* (*E. coli*) [42,44]. Advances in biotechnologies, for example codon optimization, enable the improvement of production and solubility and realize the delivery of insulin through oral ingestion of bacteria [45]. Another series of therapeutic cytokines, interleukins (ILs), have been expressed in bacteria and orally administrated for disease treatments [46,47]. For instance, IL-2 and IL-17A, both of which demonstrate their antitumor efficacies, are produced and secreted by a recombinant *Lactococcus lactis* strain [48,49]. IL-35, an anti-inflammatory cytokine, is also delivered by an engineered bacterium for the prevention and treatment of dextran sulfate sodium (DSS)-induced colitis (Figure 2A) [47]. Moreover, therapeutic proteins have been attached to bacteria via surface decoration for combination therapy [22,50]. Recently, we have exploited silk fibroin that shows anti-inflammatory effects and can target the ulcerous or damaged areas of the intestine to decorate probiotics by self-assembly on their surface (Figure 2B) [50]. Silk fibroin is co-delivered as a therapeutic protein drug to synergistically enhance the treatment efficacy in mice associated with intestinal mucositis. Meanwhile, the self-assembled silk fibroin could form an entire shell on the surface to protect the decorated probiotics from the insults in the GI tract.

Therapeutic peptides are another class of therapeutic proteins. Gastric stable pentadecapeptide, BPC-157, is reported to be able to prevent and treat GI inflammations [51]. In order to deliver BPC-157 orally, lactic acid bacteria, which are capable of producing and delivering diverse therapeutic proteins via genetic engineering [52,60,61,62,63], are used to produce BPC-157 fusing with a membrane protein. The heterologous peptide is successfully displayed on the bacterial surface and orally delivered into the host as a therapeutic agent to reduce reactive oxygen species (ROS) production [52]. Another therapeutic peptide, glucagon-like peptide-1 (GLP-1), for the treatment of non-insulin-dependent diabetes, is delivered orally by a recombinant *Lactococcus lactis* that is genetically modified with a plasmid-encoding GLP-1 cDNA [41]. In addition, antibodies and antigens have also been expressed by bacteria, which are delivered orally to elicit strong immune responses [1,10,11,63]. The soluble receptor activator of nuclear factor kappa-Β ligand (RANKL) expressed in *Lactococcus lactis* exhibit the potential to act as an oral vaccine adjuvant that enhances the systemic and mucosal immune responses [64]. As an antigen, the HIV envelope protein is expressed in commensal *Streptococcus mitis*, which are co-administrated as an oral vaccination to induce both salivary and systemic antibody responses and develop antigen-specific systemic T cell tolerance [65].

### 2.2. Nucleic Acids

Nucleic acids, as a type of macromolecular therapeutic agents, are a series of functional DNA and RNA [19]. They have been widely applied for gene therapy in different forms including short-interfering RNAs, DNA/RNA vaccines, and genetic pharmacology [66]. In general, nucleic acid drugs include aptamers, interfering RNAs, antigens, ribozymes, and antisense nuclear acids. Nucleic-acid-based therapeutics exhibit the potential to treat a number of diseases by correcting the abnormal expression of specific genes, by virtue of their characteristic of high specificity to target genes [19]. However, due to the presence of multiple biological barriers including enzymatic barriers, mucus gel barriers, and cell membrane barriers in the GI tract, the oral delivery of nucleic acid drugs suffers from similar challenges to therapeutic proteins/peptides [1,10].

**Figure 3:**
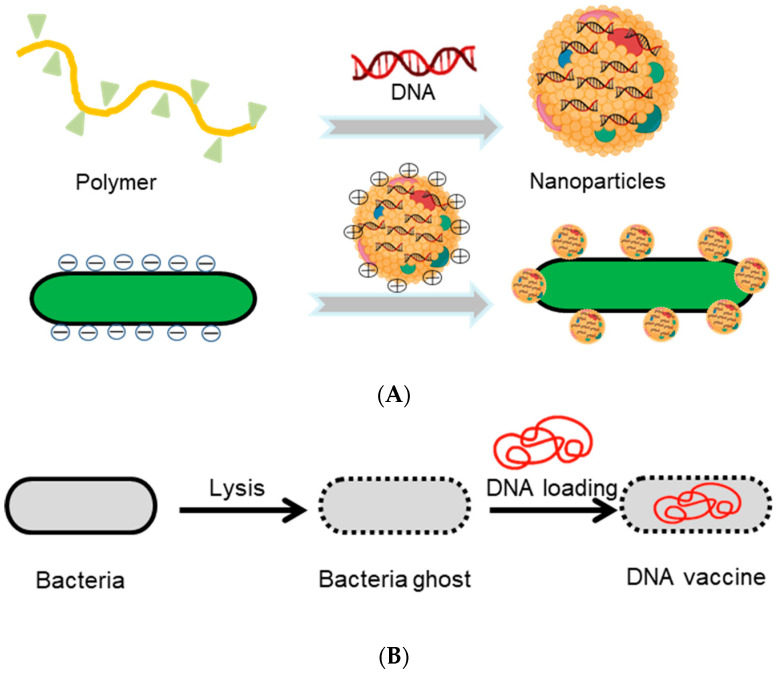
Engineered bacteria for the oral delivery of nucleic acids. (**A**) DNA nanoparticle-decorated bacteria as an oral vaccine for cancer therapy. Adapted from [74], published by American Chemical Society, 2015. (**B**) DNA vaccines orally delivered by bacterial ghost for HIV. Adapted from [78], published by Elsevier BV, 2012.

To overcome obstacles encountered in the oral administration of nucleic acids, numerous strategies that can address instability resulted from enzymatic degradation and side effects caused by anionic charges and enhance the oral bioavailability have been developed [67,68]. It is worth noting that some of them have entered into clinical trials [53,69]. The main approaches developed for nucleic acid delivery can be categorized into non-virus (such as liposomes, polymer vectors, and plasmid DNA) and virus vectors (such as adenovirus and retrovirus) [68,70]. As an alternative, bacteria have been extensively engineered to deliver nucleic acids, such as plasmid DNA, aptamers, and DNA vaccines for treating IBDs and colon cancer [1,71,72]. Non-pathogenic bacterial species are considered as a promising approach to drug delivery in both forms of intravenous injection and oral administration. For example, the human Elafin gene, encoding Elafin that is absent in mucosa of IBD patients, has been delivered via a plasmid vector inside oral probiotics for inflammation inhibition and intestinal flora regulation [54]. Additionally, *Lactococcus lactis* has been developed as a therapeutic microdevice to deliver *IL-10* gene that encodes an immunomodulative protein for IBD treatment [73]. With the merit of in situ production, IL-10 is able to avoid degradation from intraluminal harsh environments and maintain their native therapeutic activities [73]. Meanwhile, pathogenic bacteria, such as *Clostridium* species, *Salmonella Typhimurium* (*S. Typhimurium*), and *Listeria monocytogenes*, have been applied for targeted tumor killing and oral vaccines [20,63,74,75]. Since these invasive species can deliver heterologous genes into tumor cells for expressing antitumor drugs, their tumor killing efficacies have been improved significantly [75,76]. Furthermore, chemically modified bacteria have been investigated for the oral delivery of DNA. For instance, Hu and colleagues have developed an engineered bacterium that is anchored with cationic DNA nanoparticles on the surface (Figure 3A) [74]. Equipped with protective DNA nanoparticles layers on the surface, bacteria succeed in escaping phagosomes and remain intact and active after exposure to the GI tract. The delivered DNA vaccines are able to encode vascular endothelial growth factor receptor 2 (VEGFR2) and activate T cells, showing significant inhibition against tumor growth [74]. Additionally, bacterial ghosts, empty bacterial envelopes that lack infectious abilities but display bio-adhesive properties, have been explored as vectors for the delivery of DNA drugs [20,77]. In order to induce peripheral and mucosal immune responses, Wen et al. have delivered HIV-1 gp-140 DNA vaccine through *S. typhimurium*-derived bacterial ghosts, which could activate antibody responses via the TLR4 pathway (Figure 3B) [78].

### 2.3. Polysaccharides

Polysaccharides, a sort of biological macromolecules produced by plants, animals, and microorganisms, refer to a class of therapeutic agents containing a glycan molecular skeleton or derived from carbohydrate compounds and their derivatives, such as glycoproteins, proteoglycans, glycolipids, lipopolysaccharides, and glycosides. The chains of polysaccharides with a complex structure on the cell surface play critical roles in a variety of physiological and pathological processes in organisms. However, due to a higher degree of the complexity and diversity of the structure compared to those of proteins and nucleic acids, progresses achieved in polysaccharide-based macromolecular drugs lag far behind. In recent years, with the developments of glycomics and glycobiology as well as the technologic improvements in separation and purification, structural characterization, and qualitative and quantitative analysis, more polysaccharides with excellent pharmacological activities have entered the vision of scientists and pharmacists. Polysaccharide drugs have become an important part of drug discovery and development in recent years. In this section, we focus on the bacteria-mediated oral delivery of therapeutic polysaccharide agents.

**Figure 4:**
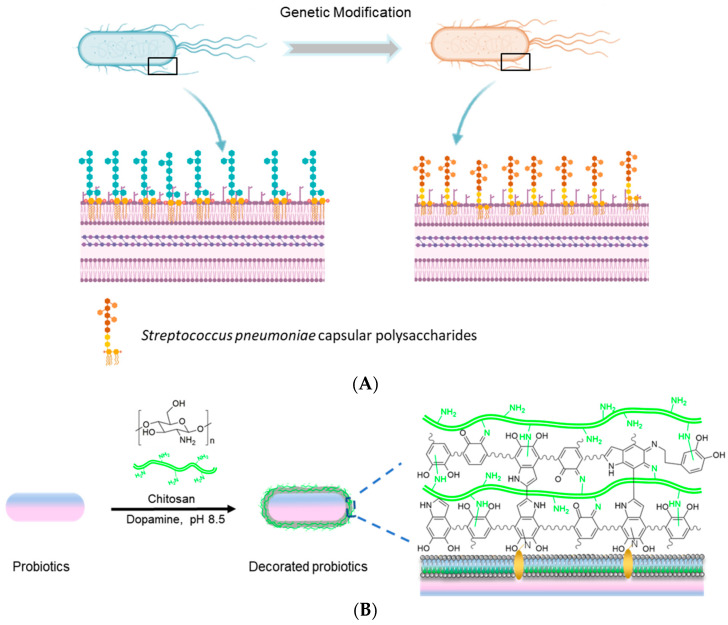
Engineered bacteria for oral delivery of therapeutic polysaccharides. (**A**) Oral delivery of *Streptococcus pneumoniae* capsular polysaccharides produced by recombinant *attenuated Salmonella*. Adapted from [82], published by National Academy of Sciences, 2021. (**B**) Attachment of chitosan on the surface of probiotics for enhanced oral delivery and biotherapy. Adapted with permission from [86]; published by Wiley-VCH, 2021.

Both lipopolysaccharides (LPS) in bacterial cell walls and capsular polysaccharides attached on the surface of the bacterial cell wall are the interface of bacteria to interact with surrounding environments. These polysaccharides are associated with the bacterial virulence and immune regulation of the host. Benefiting from their immune activation abilities, bacterial lipopolysaccharides and capsular polysaccharides have been utilized as oral vaccines [79,80]. For example, *E. coli* has been glycol-engineered as a polyvalent pneumococcal bioconjugate vaccine by using recombinant techniques and established as a robust platform for the development of bioconjugate vaccines to prevent and treat numerous pathogenic infections [81]. The immunogenic and protective pneumococcal bioconjugates produced by *E. coli* are able to endow mice with high levels of bactericidal killing activities, suggesting a promising potential of bioconjugate vaccines to treat many severe pathogenic infections. Later, Su et al. have synthesized a *Streptococcus pneumoniae* (*S. pneumoniae*) capsular polysaccharide via recombinant attenuated *Salmonella*, which have been delivered orally as a vaccine against *S. pneumoniae* infection (Figure 4A) [82]. On the other hand, various polysaccharides, such as chitosan, cellulose, inulin, and alginate, have been utilized for probiotic encapsulation for colon-specific drug delivery, due to their biocompatibility and biodegradability [14,83,84,85]. With the help of chitosan that inhibits bacterial infection and accumulation in disease sites in the host, we have fabricated a robust platform of multimodal probiotics by decorating bacteria with dopamine and chitosan (Figure 4B) [86]. Given the hybrid dopamine and chitosan coating on the probiotic surface, the bioavailability of decorated probiotics in the gut increased to more than 30-times higher, and their accumulation in the inflamed tissue is improved by 4 times. Compared to non-encapsulated bacteria, the treatment efficacy of the coated probiotics is validated to be strikingly enhanced in colitis mice.

## 3. Biomedical Applications of Oral Bacterial Microdevices

Thanks to close connections between bacteria and the host, numbers of bacteria have demonstrated their advantages in immune regulation, homeostasis maintenance, and host health [26]. On the other side, speedy developments in synthetic biology technology and nanotechnology endow bacteria with both programmable endogenous and exogenous functions, such as chemotaxis, biomacromolecule secretion, loading of synthetic substrates, which outperform conventional diagnostics and therapeutics in a range of diseases [25]. In this section, the biomedical applications of oral bacterial microdevices are summarized, and their current status and future prospect are also discussed.

### 3.1. Diagnosis

Living bacterial cells perform biological functions by sensing and responding to different signal molecules in surrounding environments, such as nutrients, metabolites, cytokines, and biological stimuli [87,88]. Taking advantages of genetic engineering technologies, synthetic gene circuits have been created in both prokaryotes and eukaryotes, which are able to respond to specific signal molecules and continuously produce measurable unique biomarkers or therapeutic macromolecules in target sites [27,55,56,89]. By synthetic gene circuits, diagnostic bacteria can readily record and quantify the process of measurement, which cannot be achieved by conventional test strategies [89].

**Figure 5:**
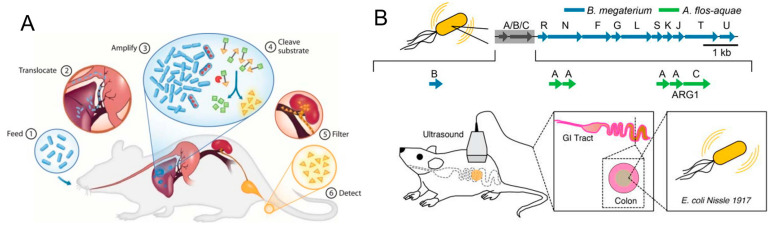
Programmed bacteria for diagnosis and bioimaging. (**A**) Genetically modified probiotics for noninvasive cancer diagnosis. Adapted with permission from [27]; published by BioMed Central, 2015. (**B**) Ultrasound imaging of engineered bacteria-carrying acoustic reporter genes in the gastrointestinal (GI) tract. Adapted with permission from [34]; published by Nature Research, 2018.

To diagnose gut inflammation and colon cancer via oral administration, bacterial strains have been engineered to sense disease markers by using a gene circuit. For example, Lee et al. have equipped *E. coli* with a responsive genetic circuit and an optimized nitrate biosensor, which could simultaneously detect thiosulfate and nitrate biomarkers [55]. Similarly, Naydich et al. have developed an *E. coli* memory circuit and identified a wide variety of new responsive bacterial biosensor triggers from inflamed gut [56]. They illustrated that this noninvasively engineered bacterial biosensor is able to report transient molecules and observe the inflammation in mouse gut for over six months. The results demonstrated that genetic engineered bacteria could be used as new strategies for the non-invasive diagnostics of inflammation diseases. Owing to the unique ability to selectively home in tumors, bacteria have been also investigated to detect cancers. For example, Danino et al. have programmed probiotics as a PROP-Z diagnostic platform for the non-invasive detection of metastatic cancer, which is difficult to be detected by conventional imaging (Figure 5A) [27]. After oral administration into the GI tract, the programmed bacteria enter metastatic tumors in the liver and the expressed specific enzyme green LacZ is cleaved into red and yellow substrates by injection with LuGal. The yellow substrate is filtered by the renal system, and the released luminescent molecules could be detected and quantified sensitively in urine. Moreover, they have successfully detected metastases from colon, lung, ovary, and pancreatic tumors in the liver by the oral delivery of these programmed probiotics. The application of bacteria-based oral microdevices for disease diagnosis, particularly for early cancer detections, is able to extend patient survival time significantly in a more effective and less painful way.

### 3.2. Bioimaging

Monitoring bacterial colonization and bioactivities in the intestinal tract is fundamental and urgent, given the important roles of the gut microbiome in regulating human health [90]. By means of the rapid blossom of genetic manipulation technology, bacteria have been genetically modified to deliver biomacromolecules orally for bioimaging. A series of conventional optic reporter genes, such as GFP and mCherry, has been utilized for the in vivo imaging of bacteria colonizing in the gut [91]. However, these optic reporters have limitations in deep tissues due to complex and harsh environments in the intestinal tract and the limited light penetration depth [34,92]. In order to improve bioimaging performance in deep tissues, researchers have developed acoustic reporter genes encoding gas-filled proteins for assembling gas vesicles, which allow the bacteria to be imaged by ultrasound with characteristics of deep tissue penetration and high spatial resolution [92,93]. For example, Bourdeau and his colleagues have designed an acoustic reporter gene based on the microbubbles of some aquatic photosynthetic organisms (Figure 5B) [34]. With the transformation of acoustic reporter genes, *E. coli* successfully expresses gas-filled protein and generates nanosized gas vesicles, which can be imaged by ultrasound when the bulk density is less than 0.01%. Furthermore, the authors have optimized the reporter genes with different acoustic characteristics, by which they have successfully detected bacteria in the host GI tract with a resolution less than 100 microns. Obviously, this technology enables the observation of the gut microbiome in vivo and contributes to the development of multimodal bacteria-mediated diagnosis and treatment.

### 3.3. Disease Treatments

The human gut microflora consisting of a huge community of microorganisms and thousands of bacterial species plays vital roles in nutrient metabolism, host immunoregulation, defense against pathogens, and maintenance of intestinal barrier integrity [90]. Imbalances of the gut microbiome are associated with a variety of diseases, such as GI infectious diseases and cancers [94,95,96,97]. Thus, to prevent and treat dysbacteriosis-related diseases, it is quite essential to positively modulate the symbiosis and composition of the gut microbiota.

#### 3.3.1. Intestinal Infectious Diseases

**Figure 6:**
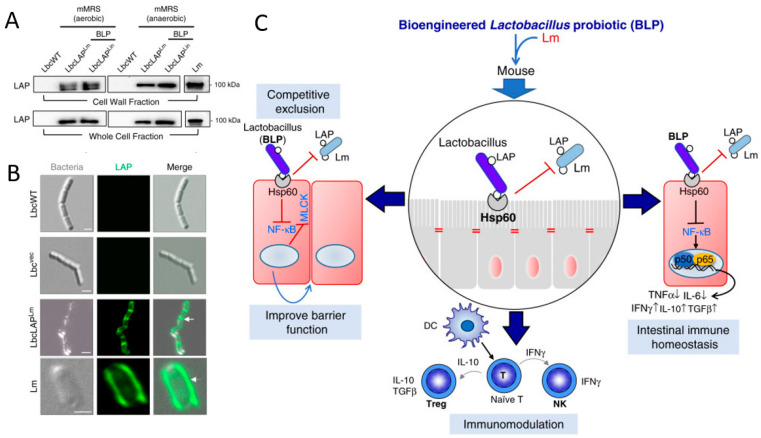
Engineered *Lactobacillus* for treating *Listeria* infection. (**A**) Detection of the expression of LAP from *Listeria* by western blot, showing LAP presenting in both the cell wall and whole-cell fractions of engineered Lactobacillus strains. (**B**) Immunofluorescence images of LAP expressed successfully on the surface of engineered *Lactobacillus*. (**C**) Schematic illustration of the mechanism for the protection effect of engineered *Lactobacillus* against *listeriosis*. Adapted with permission from [57]; published by Springer Nature, 2020.

**Figure 7:**
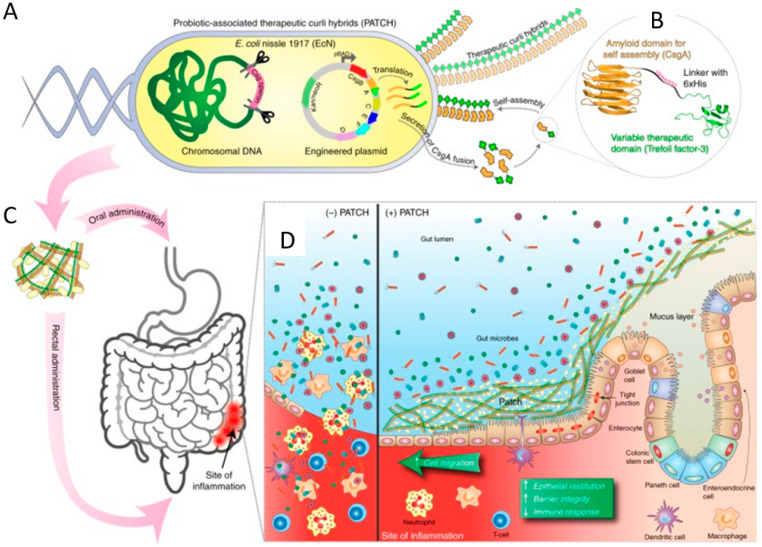
Bioengineered EcN for inflammatory bowel disease (IBD) treatment. (**A**) Self-assembly of modified curli into fibrous matrices. (**B**) Structure of curli fiber protein CsgA (yellow) fused with the trefoil factor domain (green). (**C**) Oral delivery of engineered probiotics to the site of inflammation in the GI tract. (**D**) Schematic illustration of the protective mechanisms of engineered probiotics in IBDs. Adapted with permission from [58]; published by Frontiers Media S.A, 2017.

Microbiota transplantation, including fecal microbiota transplantation (FMT) and orally delivered bacteria carrying therapeutic biomacromolecules, is an effective approach to restoring the homeostasis and health of the intestine [98]. Here, we focus on the treatment of intestinal infectious diseases by functional biomacromolecules that are orally delivered in forms of engineered bacteria (Table 1). Owing to the unique properties of bacteria, engineered bacteria are designed to express specific therapeutic biomacromolecules that can prevent and treat human diseases. Actic acid bacteria, e.g., *Lactococcus* and *Bifidobacterium*, are eatable probiotics that have been used for yogurt preparation over centuries [99]. In addition to the ability to manipulate microbiome in the intestinal tract, actic acid bacteria have been intensively investigated as an alternative for intestinal disease prevention and treatment, due to their advantages in safety, manipulability, accumulation in the GI tract, and pathogen inhibition ability [100]. With an aim to improve their anti-inflammatory property and therapeutic efficacy, genetic engineering has been performed to express a series of heterologous biomacromolecules such as therapeutic proteins/peptides of medical interests, including IL-10, IL-35, Elafin, and adhesive proteins of pathogenic bacteria [47,54,57,73]. For example, *Lactococcus fermentum* I5007 is engineered to express superoxide dismutase that exists in other bacterial species and applied for treating trinitrobenzene sulfonic acid-induced colitis by protection against ROS and inhibiting the NF-κB pathway [101]. In addition, Drolia and colleagues have camouflaged *Lactobacillus casei* by expressing the characteristic adhesive protein of Listeria (LAP) on the bacterial surface (Figure 6A,B) [57]. With the presence of LAP on the surface, the engineered *Lactobacillus* occupies LAP receptors and excludes *Listeria* competitively. In consequence, the engineered *Lactobacillus* reduces *Listeria* infection by the immune regulation mechanism of probiotics and the increased “competitiveness” enabled by bioengineering (Figure 6C). Inspired by the promising results of the recombinant actic acid bacteria in treating intestinal diseases, some of them have enter into preclinic trials [102]. *E. coli* Nissle 1917 (EcN) is another well-known probiotic bacterium, which is reported to effectively inhibit pathogenic bacteria in the gut and be engineered variously for enhancing treatment efficacies of intestinal diseases. To promote the therapeutic effects in IBD treatment, EcN is modified to express bioengineered curli fibers protein, which is able to form fibrous matrices by self-assembly (Figure 7A). The bioengineered curli fiber protein is expressed by fusing with a trefoil factor domain (Figure 7B), endowing the formed curli fibrous matrices with enhanced epithelial healing ability following the oral delivery of the engineered bacteria to the inflammation sites of the intestine (Figure 7C,D) [58]. In addition, EcN is programmed to produce an anti-biofilm protein, dispersin B (DspB), which is able to disrupt the integrity of mature biofilms [59,103]. The results suggested that after oral administration, the engineered EcN shows effective elimination and prevention activities against *Pseudomonas aeruginosa* in gut infection models.

#### 3.3.2. Cancers

Cancer is considered as a leading public health problem globally, which has caused 9.6 million deaths in 2018, with a proportion of approximately 20% in all deaths [104]. Currently, cancers have been treated mostly by conventional treatment strategies, such as surgery, chemotherapy, radiotherapy, and immunotherapy. However, these traditional therapeutic methods are often inadequate to eliminate cancers effectively and completely, because of the potential of triggering intrinsic and acquired resistance as well as unavoidable cytotoxic side effects [105]. Since Coley and coworkers pioneered the use of bacteria, e.g., *S. pyogenes* expressing Coley’s toxins, to treat cancers more than 100 years ago [105,106], the door of bacteria-mediated cancer therapy has opened. Subsequently, more bacterial species, including *Bifidobacterium* spp., *Clostridium* spp., *S. typhimurium*, and *E. coli*, have been found to accumulate in tumor sites [25]. In light of their inherent capacities of breaking related biological barriers and colonizing tumor tissue, these bacterial species have been intensively attracted for cancer therapy [107,108]. Moreover, equipped with technologies progressed rapidly in gene editing and bio-interface science, bacteria have been engineered to act as vehicles for the delivery of therapeutic agents including both small molecules and biomacromolecules (proteins and DNAs) (Table 2) [20,54,64,100,109]. In this part, engineered bacteria that are capable of delivering biomacromolecular drugs orally are discussed.

**Figure 8:**
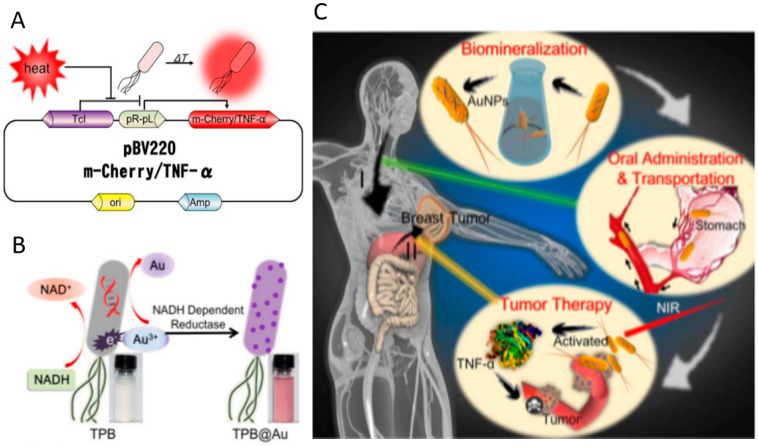
Oral delivery of bioengineered *E. coli* for cancer therapy. (**A**) Scheme of the mechanism of TNF-α expression in plasmid pBV220. (**B**) Preparation of thermally sensitive bacteria (TPB@Au). (**C**) Therapeutic effects and mechanism of the orally delivered TPB@Au. Adapted with permission from [111]; published by American Chemical Society, 2018.

To facilitate oral delivery, antitumor biomacromolecular therapeutics, such as cytotoxic proteins, cancer-specific antigens, and cytokines, have been encoded into bacteria by genetic engineering, which can produce inducibly or constantly [49,110,112,113,114]. Chung et al. have developed a bacteria-based drug delivery platform for treating colorectal cancer [110]. A lactic acid bacterium *Pediococcus pentosaceus* is modified to produce a small protein (P8) against CRC fused with a secretion signal peptide under the control of a strong inducible promoter. After oral administration into the intestine, the engineered anti-CRC therapeutic probiotics demonstrates significant anticancer efficacies in two different tumor-bearing mouse models. Combining expressing therapeutic protein TNF-α driven by a thermal-sensitive promoter and decorating with biomineralized gold nanoparticles (AuNPs) on the bacterial surface, *E. coli* MG1655 is engineered both genetically and chemically to generate a thermal-sensitive therapeutic platform termed as TPB@Au (Figure 8A,B) [111]. The engineered bacteria deliver Au nanoparticles to tumor site with the help of bacterial inherent homing capability. Upon near-infrared light irradiation, the Au nanoparticles generate heat and induce the expression of TNF-α, which could kill tumor cells (Figure 8C). In addition, researchers have generated a bacteria-based oral DNA vaccine for cancer therapy. Attenuated *Salmonella* was decorated with DNA nanoparticles that could encode autologous vascular endothelial growth factor receptor 2 (VEGFR2) on the surface (Figure 3A) [74]. The results suggested that the acid resistance of this vaccine is improved remarkably, due largely to the protection effects of the nanoparticle layers. With the assistance of bacteria that enhances the accumulation of more DNA inside tumor tissues, significantly improved therapeutic efficacy is observed in tumor-bearing mice.

#### 3.3.3. Other Diseases

In addition to intestinal inflammation diseases and cancers, bacteria have been designed to deliver therapeutic agents for other diseases, such as diabetes, obesity, HIV, and ethanol-induced liver disease (Table 3) [41,46,78]. Insulin, an essential drug for diabetes treatment, is usually delivered by subcutaneous injection as its inability to resist the strong acids and digestive enzymes in the GI tract. Thus, it is attractive to use bacteria that could be engineered to orally deliver protein/peptide therapeutics against diabetes. *Lactococcus lactis* is genetically modified to produce a single-chain insulin analog, which is able to bind and stimulate the expression of the insulin receptor [45]. Another major therapeutic drug for diabetes, GLP-1, is expressed in engineered *Lactococcus* and *Bifidobacterium longum* to enhance the efficiency of glucose control in murine models [41,115]. In addition, *Lactobacillus reuteri* is engineered to produce mouse IL-22 for treating alcoholic liver disease [46]. Previous studies demonstrated that in alcoholic liver disease models, the production of IL-22, which regulates the expression of antimicrobial C-type lectin regenerating islet-derived 3 gamma (REG3G), is reduced significantly [116]. To restore REG3G expression in the intestine, probiotic bacteria are genetically modified to orally deliver IL-22 [46]. As a result, compared to mice administered with unmodified bacteria, mice fed with engineered bacteria exhibit reduced liver damage and inflammation. These works verify that oral-bacteria-based microdevices propose an alternative to solve the problems associated with the parenteral administration of anti-diabetes drugs, such as pain, patient reluctance, and needle-related injuries and risks.

## 4. Conclusions and Future Prospects

In summary, bacteria can be easily constructed by either genetic engineering or physicochemical modification to carry various therapeutic macromolecules including proteins/peptides, nucleic acids, and polysaccharides. Given their living characteristics, such as proliferation and colonization in the gut, different bacterial species have been fabricated as versatile, yet intelligent, microdevices for the oral delivery of diverse biologicals. These living-bacteria-based microdevices have demonstrated great potential for biomedical applications including bioimaging, diagnosis, and treatment in intestinal inflammation and infectious diseases, cancers, and diabetes. Encouragingly, a few engineered bacteria have shown promising treatment efficacies and enter into clinical trials. Despite a remarkable progress has been made in this field, the oral delivery of therapeutic biomacromolecular drugs by bacteria-based microdevices faces a couple of substantial obstacles that are needed to be overcome urgently. Firstly, due to the complexity of synthetic biological techniques, many macromolecular agents cannot be expressed in their native forms by genetic modification, which suggests that the kinds of macromolecules could be orally delivered by bacteria are limited. Moreover, it is quite essential to protect bacteria from strongly acidic and digestive environments in the GI tract, with aims to increase their bioavailability and colonization. Furtherly, the disease-targeting ability of engineered bacteria is necessary to be improved. With adequate targeting ability, more bacteria can accumulate in the right sites, and hence, more therapeutic drugs can be delivered. Lastly, new mechanisms and strategies regarding increments in absorption and penetration across the intestinal barrier should be considered particularly in the delivery of biologicals into distal organs or tissues. In general, much work on bacteria-based microdevices for the oral delivery of macromolecules has been limited to in vitro or preclinical animal studies, suggesting that more efforts are needed to promote future translation of these advanced microdevices for clinical applications. However, we believe that current limitations remaining in the field are addressable considering the speedy advancements in synthetic biological methodologies, nanotechnology, and related interdiscipline. We anticipate that bacteria-based microdevices could pave an avenue for the preparation of next-generation drug carriers for the oral delivery of biomacromolecules.

## Figures and Tables

**Table 1:** Therapeutic biomacromolecules orally delivered by bacteria for treating intestinal infectious diseases.

Bacteria Species	Therapeutic Agents	Modification Strategies	Types of Therapy	Refs
*Lactococcus lactis*	IL-35	Genetic modification	Dextran sulfate sodium (DSS)-induced colitis	[32]
*Escherichia coli* (*E. coli*) Nissle 1917	Silk fibroin	Surface decoration	DSS-induced colitis	[33]
*Lactococcus lactis*	Elafin	Genetic modification	Inflammatory bowel disease (IBD)	[51]
*Lactococcus lactis*	IL-10	Genetic modification	IBD	[52]
*Salmonella typhimurium*	*Streptococcus pneumoniae* capsular polysaccharides	Genetic modification	*Streptococcus pneumoniae* infection	[53]
*E. coli* Nissle 1917	Chitosan	Surface decoration	DSS-induced colitis	[54]
*E. coli*	A responsive genetic circuit	Genetic modification	Inflammation disease diagnosis	[55,56]
*Lactobacillus casei*	Listeria adhesion protein	Genetic modification	*Listeria* infection	[57]
*E. coli* Nissle 1917	Trefoil factor	Genetic modification	IBD	[58]
*E. coli* Nissle 1917	Dispersin B (DspB)	Genetic modification	Gut infection	[59]

**Table 2:** Therapeutic biomacromolecules orally delivered for bacteria-mediated cancer therapy.

Bacteria Species	Therapeutic Agents	Modification Strategies	Types of Therapy	Refs
*E. coli* Nissle 1917	LacZ (plasmid) luxCDABE (genomic)	Genetic modification	Cancer diagnosis	[27]
*Lactococcus lactis*	IL-17A	Genetic modification	Cancer	[48]
*Lactococcus lactis*	IL-2	Genetic modification	Cancer	[49]
*Salmonella typhimurium*	DNA	Surface decoration	Cancer	[74]
*Pediococcus pentosaceus*	P8	Genetic modification	Cancer	[110]
*E. coli*	TNF-α	Genetic modification	Cancer	[111]

**Table 3:** Therapeutic biomacromolecules based on oral bacterial microdevices for treating other diseases.

Bacteria Species	Therapeutic Agents	Modification Strategies	Types of Therapy	Refs
*Lactococcus lactis*	GLP-1	Genetic modification	Diabetes	[41]
*Lactococcus lactis*	Insulin	Genetic modification	Diabetes	[45]
*Lactobacillus reuteri*	IL-22	Genetic modification	Ethanol-induced liver disease	[46]
*Salmonella typhimurium* derived bacterial ghost	gp-140 DNA	Loading	HIV-1	[78]
*Bifidobacterium longum*	Glucagon-like peptide-1 (GLP-1)	Genetic modification	Diabetes	[115]
